# Haemorrhoidal haemodynamic changes in patients with haemorrhoids treated using Doppler-guided dearterialization

**DOI:** 10.1093/bjsopen/zrab012

**Published:** 2021-04-11

**Authors:** A Parello, F Litta, V De Simone, P Campennì, R Orefice, A A Marra, M Goglia, L Santoro, A Santoliquido, C Ratto

**Affiliations:** 1 Proctology Unit, Fondazione Policlinico Universitario Agostino Gemelli IRCSS, Rome, Italy; 2 Medical Vascular Disease Clinic Unit, Fondazione Policlinico A. Gemelli IRCSS, Rome, Italy; 3 Catholic University of the Sacred Heart, Rome, Italy

## Abstract

**Background:**

Arterial hyperflow to haemorrhoids has been implicated as a possible pathophysiological co-factor in haemorrhoidal disease. The purpose of this study was to investigate how transanal haemorrhoidal dearterialization (THD) can influence haemodynamic parameters at the level of the haemorrhoidal piles.

**Methods:**

Patients with grade III haemorrhoids selected for THD between July and December 2018 were evaluated using endoanal ultrasonography and colour Doppler imaging at the level of internal haemorrhoids before and 1 year after the surgical procedure. Peak systolic velocity, pulsatility index, resistivity index, acceleration time, and end-diastolic velocity were measured, and preoperative and postoperative values compared. Symptom severity was measured using a symptom-based questionnaire (score range 0–20).

**Results:**

Of 21 patients treated, 17 completed the study. Compared with preoperative values, postoperative peak systolic velocity (mean(s.d.) 18.7(1.1) *versus* 10.3(0.4) cm/s; *P* < 0.05), pulsatility index (5.5(0.3) *versus* 2.8(0.4); *P* < 0.050), and resistivity index (1.0(0.2) *versus* 0.8(0.5); *P* < 0.050) decreased significantly, whereas acceleration time increased significantly (65.6(3.6) *versus* 83.3(4.7) cm/s^2^; *P* < 0.050); end-diastolic velocity did not change (1.9(0.2) *versus* 2.0(0.4); *P* = 0.753). Symptoms disappeared or had improved significantly in all patients by 1 year after surgery. The mean(s.d.) total symptom severity score decreased from 15.8(1.1) to 1.2(1.6) (*P* < 0.001).

**Conclusion:**

THD affects the main haemodynamic parameters at the level of internal haemorrhoids and is associated with a decrease in arterial hyperflow.

## Teaser

The purpose of this observational study is to understand how transanal haemorrhoidal dearterialization (THD) procedure, a surgical approach aimed to reduce the hemorrhoidal iperflow, can influence the haemodynamic parameters at the level of the hemorrhoidal piles.

THD procedure affects the main hemodynamic parameters at the level of internal haemorrhoids, reducing the arterial hyper-flow and justifying the use of this approach to treat haemorrhoidal engorgement and bleeding

## Introduction

Hyperflow of haemorrhoidal arteries has been recognized as an important pathophysiological phenomenon associated with haemorrhoidal disease^1–3^. Haemorrhoidal dearterialization has been proposed to allow the reduction of blood hyperflow to haemorrhoids. This minimally invasive approach offers a therapeutic option for patients with haemorrhoidal disease without any tissue excision. Following encouraging clinical results over the years, the target area of the procedure has been studied with the aim of mapping the haemorrhoidal arterial pattern at the level of the low rectum. It has been demonstrated that almost all of the haemorrhoidal arteries in the terminal 2 cm of lower rectum are superficial and can be found in the submucosa^4^. This study allowed a modification of the traditional technique, with the aim of optimizing the reduction in blood hyperflow to the haemorrhoidal plexus^5^^,^^6^. This approach can also provide rectal mucopexy for coexisting haemorrhoidal prolapse. The technological evolution of devices specifically designed for this procedure over the years has made it possible to treat patients with clinically relevant haemorrhoidal prolapse that required either sectorial or full-circumference repair, even in the setting of third- or fourth-degree haemorrhoidal disease. The advantage of this combined approach is reduction of symptoms associated with haemorrhoidal engorgement by reducing blood hyperflow through dearterialization, and repairing the haemorrhoidal prolapse by means of mucopexy without any tissue excision.

Despite good clinical results, the haemodynamic impact of this technique on haemorrhoids has not been investigated. The present study aimed to investigate haemorrhoidal haemodynamics in patients with grade III haemorrhoids undergoing transanal haemorrhoidal dearterialization (THD) using colour Doppler imaging, and to evaluate the severity of symptoms after surgical treatment.

## Methods

The study was approved by the local institutional ethical committee, and patients were informed about and agreed on the aims, investigations, surgical procedure, and follow-up timing and modalities.

### Patients

All patients with grade III haemorrhoids selected for THD between July and December 2018 were screened for inclusion. Inclusion criteria were: age between 18 and 65 years, grade III haemorrhoids with circumferential prolapse, symptomatic for at least 12 months, and resistance to conservative therapy. Exclusion criteria were: chronic inflammatory bowel disease, antiplatelet and/or anticoagulant therapy, vasculopathies, previous colonic and/or rectal resections, previous proctological surgery, previous pelvic radiotherapy, severe constipation or diarrhoea, and intractable irritable bowel disease.

Before surgery, patients underwent full physical and clinical examinations, followed by completion of a symptom-based questionnaire, recording the frequency of bleeding, prolapse, manual reduction, discomfort/pain, and impact on quality of life, with responses graded from 0 (no symptoms) to 4 (daily presence of each symptom(s). The total score for all five parameters was used to evaluate the patient’s condition; a score of 0 indicated total absence of symptoms, whereas 20 represented the worst clinical scenario^7^. Colonoscopy was undertaken according to guidelines for colorectal cancer screening. Endoanal ultrasonography (EAUS) and colour Doppler imaging were also performed using an ultrasound system (Pro-Focus Green^TM^; BK Medical, Herlev, Denmark) fitted with endoanal probes (models 2052 and 8848; BK Medical).

### Operative procedures

Under general anaesthesia, all patients underwent haemorrhoidal dearterialization and mucopexy according to the THD Doppler procedure (using THD Slide device; THD, Correggio, Italy), as described elsewhere^5^. In particular, the distal Doppler-guided dearterialization technique^6^ was used. During the procedure, six arteries were identified in the distal lower rectum by Doppler imaging, within 1–2 cm above the anorectal junction. Their position was traced as a marker point on the rectal mucosa. Mucopexies, necessary for grade III haemorrhoids, were always performed in six sectors of the rectal circumference, corresponding to sites of haemorrhoidal arteries. Each mucopexy included redundant and prolapsing rectal mucosa/submucosa within a running suture, incorporating the haemorrhoidal artery already identified and marked, and was finally extended up to the anorectal junction, excluding internal haemorrhoidal piles. The interval between mucopexies was maintained to avoid complete circumferential involvement of the perirectal venous network into the mucopexies. Knotting the stitch completed both the dearterialization (strangulating the haemorrhoidal artery into the suture) and mucopexy (plicating the prolapsing mucosa/submucosa into the knot), thus providing a haemorrhoid-saving operation. At the end of the procedure, a posterior perineal block was used to control postoperative pain. At hospital discharge, anti-inflammatory therapy, on-demand analgesic, and a diet rich in water and fibre, supplemented with oral fibre, were prescribed.

### Follow-up

Patients underwent clinical follow-up at 1, 3, and 12 months after the procedure. During the 12-month follow-up, the questionnaire concerning symptoms was readministered, and EAUS and colour Doppler imaging were repeated to assess haemorrhoidal haemodynamic parameters after operation.

### Outcome measures

During EAUS and colour Doppler imaging, the haemorrhoidal arteries identified within the internal haemorrhoidal piles were investigated based on peak systolic velocity (PSV), pulsatility index (PI), resistivity index (RI), acceleration time (AT), and end-diastolic velocity (EDV). Symptom frequency and the total score derived by the questionnaire were calculated.

### Statistical analysis

A descriptive analysis of patient characteristics was undertaken. Mean(s.d.) values were calculated for all quantitative variables. Qualitative or categorical variables were described as frequencies and proportions. For normally distributed data, means were compared using the *t* test for dependent samples, whereas the Wilcoxon test was used for non-normally distributed data. All statistical tests were two-sided and performed at a significance level of α = 0.05. Data were analysed using SPSS^®^ version 17.0 (IBM, Armonk, New York, USA).

## Results

Twenty-one patients were enrolled in the study. Three patients who underwent an additional procedure (left lateral sphincterotomy owing to coexisting anal fissure) during dearterialization and mucopexy were excluded. One patient declined the follow-up re-evaluation. Therefore, data on 17 patients (11 men, mean(s.d.) age 46(9) years) who completed the follow-up programme were analysed.

All six arteries were identified successfully during operation in the entire cohort and no intraoperative complications were observed. After operation, four patients required analgesics beyond the fifth postoperative day, postoperative bleeding (not requiring surgical haemostasis) occurred in three patients, and urinary retention in one. Neither haemorrhoidal thrombosis nor long-term complications were recorded.

**Fig. 1:**
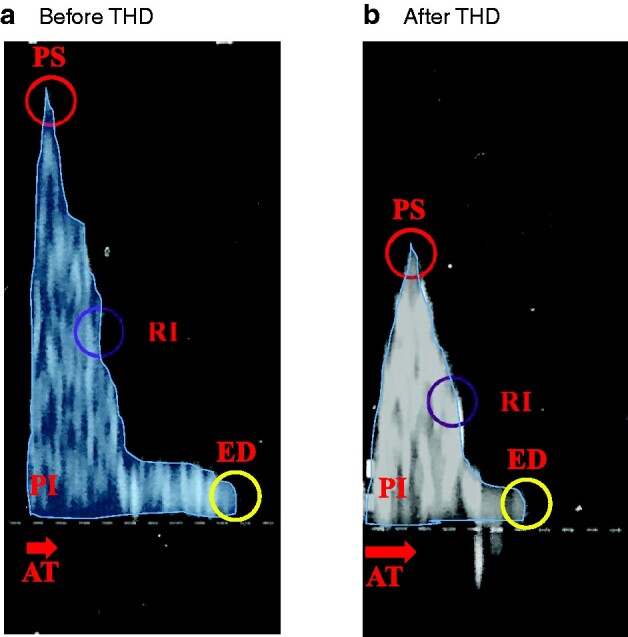
a Before and b after transanal haemorrhoidal dearterialization (THD). PS, peak systolic; RI, resistivity index; PI, pulsatility index; ED, end diastole; AT, acceleration time.

Symptoms and Doppler findings at 12-month follow-up were compared with preoperative ones (*Fig. 1*). Colour Doppler imaging, at the level of the internal haemorrhoidal piles, showed that mean PSV, PI, and RI were significantly decreased compared with preoperative values. On the other hand, mean AT increased significantly after operation; mean ED did not change (*Table 1*).

**Table 1:** Haemodynamic parameters before and after transanal haemorrhoidal dearterialization

	Before surgery	After surgery	*P*
Peak systolic velocity (cm/s)	18.7(1.1)	10.3(0.4)	<0.05
Pulsatility index	5.5(0.3)	2.8(0.4)	<0.05
Resistivity index	1.0(0.2)	0.8(0.5)	<0.05
Acceleration time (cm/s^2^)	65.6(3.6)	83.3(4.7)	<0.05
End-diastolic velocity (cm/s)	1.9(0.2)	2.0(0.4)	0.753

Values are mean(s.d.). *t test.

Symptoms disappeared or improved significantly in all patients. The mean(s.d.) total score decreased from 15.8(1.1) (range 14–18) to 1.2(1.6) (range 0–5) (*P* < 0.001).

## Discussion

The THD Doppler procedure aims to interfere primarily with arterial hyperflow to the haemorrhoids (considered responsible for pile engorgement and bleeding; treated with dearterialization) and the effects of connective degeneration (leading to prolapse; treated with mucopexy). As a cascade, the procedure should also have a beneficial effect on other symptoms of haemorrhoidal disease. The efficacy of dearterialization should be demonstrated not only by control of symptoms after surgery, but also by investigating its haemodynamic effect. Clinical results seemed sufficiently robust in showing improvement in haemorrhoidal pile engorgement, significant reduction in bleeding episodes, and very low incidence of recurrent bleeding^6–10^.

In the literature, the dearterialization technique has shown considerable variability, probably for several reasons, such as differences in devices applied, use of Doppler, presence or absence of mucopexy, number of ligatures performed, and type of stitch used. A systematic review^9^ including 2904 patients described a relapse rate of between 3 and 60 per cent, with a pooled recurrence rate of 17.5 per cent and a reoperation rate of 6.4 per cent. To improve the data quality, it seemed mandatory to standardize the dearterialization target. This study documented the benefits of identifying, with utmost precision, the location of the terminal branches of the upper haemorrhoidal arteries. Based on the results, the surgical technique was standardized in order to systematize the pathophysiological impact of this approach. The clinical results observed were consistent with recurrence of symptoms related to haemorrhoids in 9.5 per cent of patients and a reoperation rate of 7 per cent^7^. On the other hand, a systematic review and meta-analysis^10^ comparing the main surgical treatments for haemorrhoidal disease showed an advantage of THD in terms of shorter operating time, reduced pain scores and postoperative complications, but a higher relapse rate.

However, the haemodynamic modifications to the tissue target resulting from the surgical procedure remained to be demonstrated. These modifications were analysed in the present study. Measuring haemodynamic parameters 1 year after surgery allowed demonstration of a well stabilized haemodynamic situation. The decreased PSV, PI, and RI may be explained by the preoperative arterial flow (supposedly excessively high before operation) being greatly reduced after the THD Doppler procedure. The second effect concerned the AT, which significantly increased after operation. In terms of haemodynamics, this means that, for the blood, the time to reach the PSV was longer than that before surgery. All these haemodynamic effects can by accounted for by modifications from preoperative arterial hyperflow to significantly reduced flow. Correlating these data with the clinical effects of the operation (very low-grade symptoms in all treated patients at 12 months after operation), they clearly demonstrate the impact of dearterialization on symptoms of haemorrhoidal disease. The last relevant result produced by the THD Doppler procedure was its lack of effect on the EDV, which remained very similar to the mean preoperative value. The THD Doppler procedure did not provide a significant haemodynamic obstacle to blood circulation at the venous network level, consequently avoiding an increased risk of haemorrhoidal thrombosis, as confirmed in all patients in this study.

The THD Doppler procedure offers reliable efficacy in achieving haemorrhoidal dearterialization, thanks to effective ligation of the arteries leading to successful reduction in arterial hyperflow. This study has elucidated the haemodynamic effects of arterial ligation and justifies the use of this approach to treat haemorrhoidal engorgement and bleeding, leaving the haemorrhoidal piles fully viable with intact anatomy and physiology.

*Disclosure:* The authors declare no conflict of interest.
